# A Dynamically Reconfigurable Autonomous Underwater Robot for Karst Exploration: Design and Experiment

**DOI:** 10.3390/s22093379

**Published:** 2022-04-28

**Authors:** Tho Dang, Lionel Lapierre, Rene Zapata, Benoit Ropars, Guillaume Gourmelen

**Affiliations:** 1 Laboratory of Informatics, Robotics and MicroElectronics (LIRMM) (UMR 5506 CNRS-UM), Université Montpellier, 161 rue Ada, CEDEX 5, 34392 Montpellier, France; zapata@lirmm.fr (R.Z.); gourmelen@lirmm.fr (G.G.); 2 Reeds Company, 199 rue Hélène Boucher, 34170 Castelnau-Le-Lez, France; ropars@lirmm.fr

**Keywords:** autonomous underwater robot, reconfigurable underwater robot

## Abstract

This paper presents the design and experiment of an autonomous underwater robot which can change the geometric configuration of its actuators, according to mission requirements or environmental constraints. The robot consists of two subsystems: forward part with three thrusters and backward part with four thrusters. The position and orientation of these thrusters can be dynamically changed during missions. Being different from most of other reconfigurable underwater robots which were designed as linked-modules, our robot has a unified design. It is suitable for specific mission in confined environments (e.g., karst exploration) in which the robot has to modify its shape to go through a narrow section or align the most part of its thrusters in the direction of a strong current, for examples. The design procedure, from hardware to software, of the robot is presented and experimental results are shown to demonstrate the versatility of the robot. Furthermore, the discussion and comparison between our robot and other underwater robots with adaptable actuation geometry are presented to highlight advantages of our design. Finally, the idea of using our robot for classic docking problem, which has some common features with karst exploration requirements in using dynamically reconfigurable robots, is discussed.

## 1. Introduction

### 1.1. Karst Exploration

Karst generally comprises a network of underground natural conduits, resulting from the dissolution of soluble rocks, limestone, dolomite, and gypsum. These aquifers drain groundwater on a large scale from their inland catchment basin to their marine exsurgences. They supply drinking water to millions of people worldwide and, during heavy rainfall, may host violent transfers of charge that can cause dramatic and sudden floods in fragile and unpredictable areas. The urgent need for management tools of underground resources requires a precise knowledge of the underlying conduit network, in terms of position, depth, geomorphology, and seasonal and episodic dynamics. Exploitation of this resource requires precise drilling, which must penetrate these conduits in a region with an appropriate morphology (pumping chamber), in order to reply to the pumping demand, also considering the seasonal variability of the resource availability and quality. Hydrogeological risk management requires having a precise knowledge of the hydrosystem dynamics, running models in order to predict floods occurrence, or afford this underground network with a dam flood control role [1]. It is thus of major importance to obtain reliable information about the position, geometry, and dynamics of these karstic networks along their entire development, from inland to marine resurgence. This is a crucial and urgent issue for public authorities in charge of prospection, protection, and active management of the groundwater resource in karstic regions.

### 1.2. Reconfigurable Robots

In robotic fields, reconfigurable robots have been an attractive area because of their versatility. They can change their shape or configuration corresponding to specific mission requirements; therefore, the building cost may be reduced with one robot doing several works. Moreover, reconfigurable robots can be applied in complex tasks requiring adaptive configurations such as karst exploration or space applications. For instance, a clear operational reason for reconfigurable robots is to minimize power consumption. Robustness is also an advantage of reconfigurable robots in virtue of its flexibility. Readers can read the overview of these questions and other issues of modular self-reconfigurable robot system in [2,3].

In robot manipulators, the idea of reconfigurable robot was initially driven by manufacturing industry as shown in [4,5,6,7]. This has been extended to other fields of robotics such as land-based and underwater robot areas. The prominent idea for reconfigurable robot is a modular design concept in which the robot can connect or disconnect its corresponding modules [8,9]. For instance, a modular reconfigurable robot with perception-driven autonomy was proposed in [10], where the robot is able to complete complex tasks by reactively reconfiguring to meet the perceived environmental information. A floor cleaning robot with a reconfigurable mechanism was introduced in [11], where the robot reconfigures its morphology in response to its perceived environment to maximize coverage area. A reconfigurable snake robot was presented in [12]. The snake robot was also designed using modules; however, the robot can transform to various configurations without the rearrangement of modules. A gait planner is used to switch between configurations: snake gait, transforming gait, and walking gait.

In the underwater field, a guidance and control method for a reconfigurable unmanned underwater vehicle was introduced in [13]; however, the reconfigurability of the robot is based on the thruster’s redundancy management, where the thruster’s configuration is fixed. In [14], a reconfigurable robotic fish with undulating fins was developed; however, it is not a dynamically reconfigurable capability, just reconfiguring design parameters to achieve another version of the robot. Another reconfigurable robotic fish was introduced in [15]. The robot was designed in a modular way in order to build different morphologies, before operation. Reconfigurable magnetic-coupling thrusters for AUVs were introduced in [16,17,18,19]. The main idea here is based on the use of two coupled magnetic elements on which the thrusters are mounted and allowing for dynamically changing the direction of their thrust. Hence, the number of actuators is increased (doubled), as well as the cost of the system. Moreover, the magnetic filed between coupling magnets is easily disturbed by the metal parts of the robot structure. The idea of using magnetic coupling to build versatile a thruster configuration is also used in [20]. Reconfigurable AUV for Intervention (RAUVI project) was presented in [21,22,23]. This is an autonomous underwater robot equipped with one manipulator that allows the robot to perform manipulation tasks. The robot, inherited from Girona 500 AUV [24], is statically reconfigured with respect to different tasks. A prototype of a reconfigurable underwater robot with a bioinspired electric sense was introduced in [25]. The robot was designed as modules that can be detached or attached in order to adapt its configuration. In [26], a dynamics and control approach for modular and self-reconfigurable robotic systems was presented. Several benchmark examples are used to evaluate different configurations. In robotic systems, the reconfigurability can be found in any stage of the robotic architecture, from software to hardware, concepts of reconfigurable autonomy can be found in [27]. Nevertheless, in this paper, we only consider reconfigurability at actuation configuration. A static reconfigurable underwater robot, named *SeaDrone*, was introduced [28]. Four configurations of the robot corresponding to four underwater tasks were shown; however, this is performed statically before mission execution. A structure of a reconfigurable AUV/ROV for man–robot underwater cooperation was depicted in [29]. It can be mechanically modified with six possible layouts. The *SubSea Tech* company has been developed a reconfigurable robot, called *Tortuga*, which can change the direction of horizontal thrusters [30]. Specifically, one thruster needs one motor to change its direction in horizontal plane.

**Figure 1:**
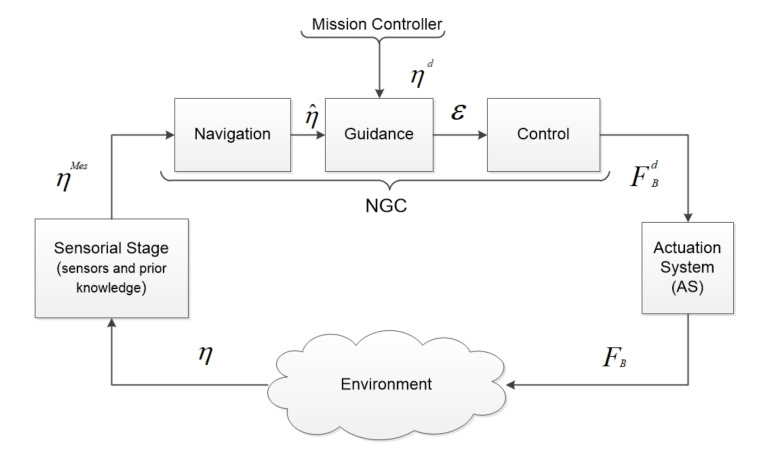
The general diagram of Sensory Navigation Guidance and Control Actuation (S-NGC-A) [31].

**Figure 2:**
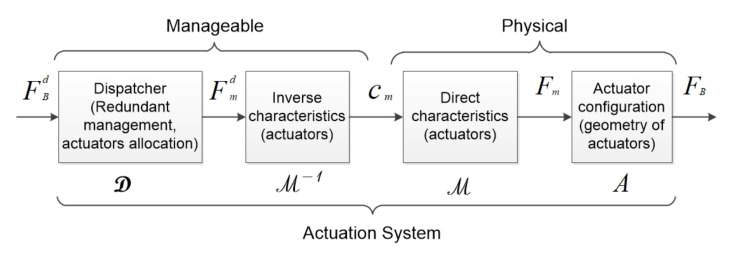
Actuation system scheme [31].

In this study, we consider the generally admitted control architecture for marine systems: NGC (Navigation Guidance and Control) scheme, around which we can reify two other modules: the Actuation System (AS) and Sensorial Stage (SS) (see Figure 1). The sensorial stage provides the necessary information ($\eta^{Mes}$) based on the sensor measurement and prior knowledge of the environment to the navigation system, which is an input for the NGC system. Inside NGC, the navigation system provides the estimation of the system’s state ($\hat{\eta }$) to the guidance system to compute an error function (ε) with respect to the reference state ($\eta^{d}$). The control system is then in charge of computing the desired body-frame action ($F_{B}^{d}$). Afterwards, the AS dispatches the desired body-frame action ($F_{B}^{d}$) to the actuators set, in terms of individual actuation thrust. The reconfigurability of the actuation geometry is implemented at AS. Referring to the AS’s structure depicted in Figure 2, based on the desired body-frame action ($F_{B}^{d}$) (the output of the controller), the dispatcher (D), considering the actuator allocation method (and eventually, redundancy management), computes the desired actuator vector ($F_{m}^{d}$) that each actuator has to produce. The inverse actuator characteristics are then taken into account to compute the actuator inputs ($c_{m}$) (classically PWM—Pulse Width Modulation). Once applied, $c_{m}$ produces actual actuator vector ($F_{m}$). The resulting vector $F_{B}$ is produced according the actuator’s configuration (A), which changes in function of the actuator’s geometry.

### 1.3. Karst Exploration with Robots

Exploring a confined environment, e.g., karst, cave, or shipwreck, is particularly challenging because of the chaotic nature of the environment in terms of geomorphology and the resulting hydrodynamics effects. This yields the need for a flexible robot that can modify its shape and actuation configuration to dynamically adapt to environmental conditions. For instance, the robot should have a compact and slender shape (torpedo-like configuration) to cross narrow sections (i.e., narrow galleries) with strong current, isotropic configuration for station-keeping, and to be capable of rotating about any axis for localized inspection and data collection. A dynamically reconfigurable robot can minimize energy consumption and be more robust thanks to the flexibility of its configuration. Indeed, given a task, this robot can modify its configuration to minimize an energy cost function. For instance, the robot has to carry out a mission such as diving to a desired depth, following a path, and rotating about several axes to observe the environmental region of interest. For a fixed-configuration robot, the controller is designed specifically to this configuration (under-actuated or fully/over-actuated system). In contrast, a dynamically reconfigurable robot can change its configuration with respect to the specific mission to reduce the efforts to achieve the control objective; therefore, a cost function can be included in the design of control strategy or control allocation method to minimize the energy consumption. Moreover, the reconfigurability allows us to optimize the actuators geometry in function of the control demand and the actuation inputs in function of some criteria, e.g., energy, reactivity. Motivated by this context, the paper presents a dynamically reconfigurable AUV, called an Umbrella Robot (UR), which can modify its actuation configuration with respect to different tasks. In fact, our robot has seven thrusters whose directions and positions can be adjusted during its operation, using two added actuators. The novelty in our research is to propose a new mechanism for a dynamically reconfigurable robot, which is different from others in the literature. In fact, other robots were designed for changing the configuration statically or connecting/disconnecting their modules thanks to the module-linked design. Our idea stems from a unified mechanism that can change the robot’s configuration dynamically. Moreover, in our design, only two added motors can change the direction and orientation of all thrusters of the robot. This means that less actuators are needed and more acting abilities are achieved. The main contributions of the paper are described as follows:

1. A complete design (hardware and software)—a dynamically reconfigurable AUV.
2. An analysis of the reconfigurable capacity of the robot.
3. A presentation of experiments to demonstrate the robot’s performance.
4. A comparison between our robot’s design and others, and propose an application case—docking problem.

The rest of the paper is organized as follows: The design procedure is presented in Section 2. The reconfigurable capacity is analyzed in Section 3. Demonstration experiments are shown in Section 4. The comparison between our robot and others is discussed in Section 5, and an application case, i.e., the docking problem, is also mentioned in Section 6. Finally, conclusions and future works are discussed in Section 7.

## 2. Design

### 2.1. General View

**Figure 3:**
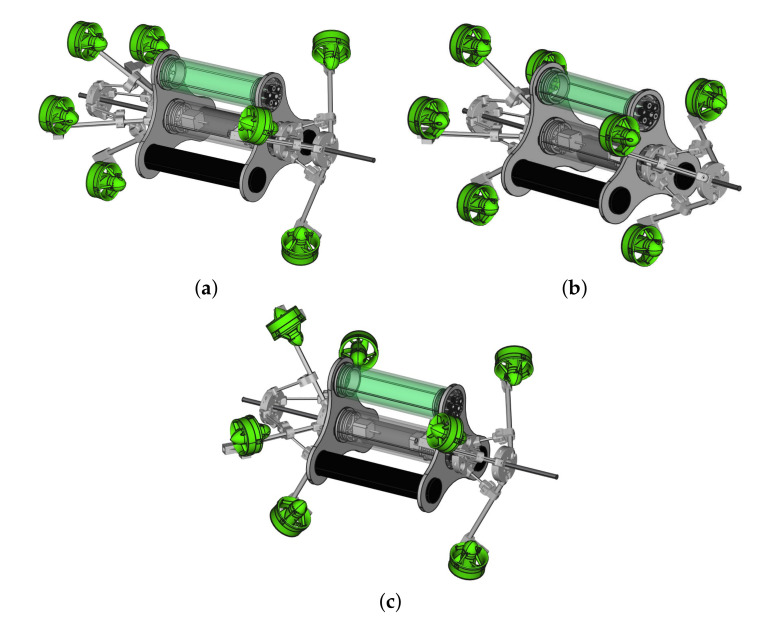
The 3D model of Umbrella Robot. (**a**) Umbrella robot in open-forward and close-backward. (**b**) Umbrella robot in close-forward and close-backward. (**c**) Umbrella robot in open-forward and open-backward.

A general view of the UR system is shown in Figure 3 in which the robot is shown with different configurations, i.e., forward thrusters in the *“open”* state in Figure 3a, *“close-close”* state for forward and backward thrusters as in Figure 3b, and *“open-open”* state (forward and backward sides) in Figure 3c. The robot carries seven thrusters: four thrusters in backward side and three thrusters in forward side. The main body of the UR is built by two tubes in which one is used for central processor and the other holds two DC motors (Direct Current motors) connected to two threaded rods (forward and backward sides), which are used to change configurations. Two battery packages (black tubes in Figure 3) are used for the robot. Waterproof cables are used for communicating between parts of the robot.

### 2.2. Hardware Architecture

**Figure 4:**
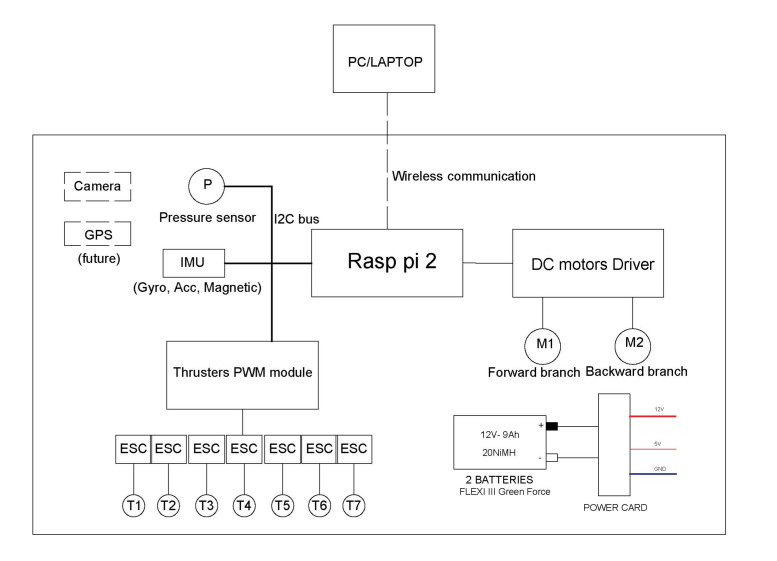
The principle diagram of UR.

We describe the hardware architecture of the UR in this section. The processor of UR is a Raspberry Pi 2. Raspberry Pi has many merits in computational capability and extensibility. The robot is equipped with a pressure sensor and an IMU sensor. One camera and six echosounders will be installed in the future. The thrusters of the robot are controlled by a PWM module, which communicates with Raspberry Pi using the *I2C* protocol. Two DC motors with encoders are used to actuate the two threaded rods and to change the orientations and positions of thrusters. The principle architecture of UR is illustrated in Figure 4. Electronic Speed Controller (ESC) is provided for each thruster. Two DC motors are controlled by DC motor drivers. Battery packages with power converter card can supply electric power with several voltage levels for the whole robot.

### 2.3. Software Architecture

This section presents the software architecture of the robot from class modeling to dynamic state machine modeling. Because of the limitation of pages, some diagrams of the software design process are not given; for instance, the dynamic interaction modeling and integrated communication diagram.

#### 2.3.1. Static and Object/Class Modeling

**Figure 5:**
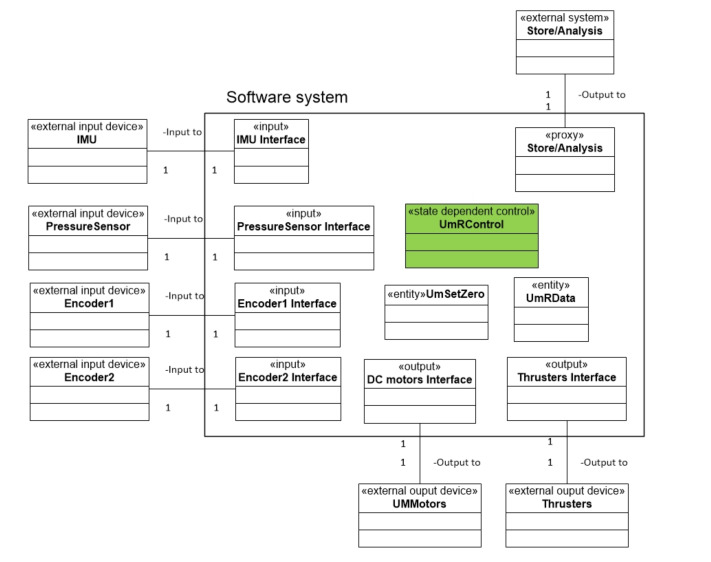
The object structuring diagram of UM Robot.

The object structuring diagram is shown in Figure 5. The software system includes four input classes, two output classes, a proxy class, two entity classes, and one state dependent class. The input classes, i.e., the IMU, pressure sensor, and two encoders, receive sensory data from sensors. The output classes, including DC motors and thrusters interfaces, communicate with the DC motors and thrusters. Two entity classes, i.e., UMSetZero and UmRData, are used to set up zero point (for changing UR’s configurations) and to store operation data, respectively. One proxy class transfers data to an external device, such as a personal computer or laptop.

#### 2.3.2. Dynamic State Machine Modeling

The dynamic state machine diagram describes the robot’s states during operations. The state machine follows the states of the robot as its transitions from the idle state to other states. The states are determined by following the use cases (turn on power/automatic, turn on power/manual, turn off, upload data). In the paper, only the dynamic state machine of *Turn on power/automatic* use case is shown as follows:

(1) **Idle**: This is the initial state, in which the robot is idle, waiting for a specific time before starting missions (this time is saved for putting the robot into the water). In this state, the robot sets its umbrellas (i.e., forward and backward sides) into zero point and checks all initial conditions.
(2) **Starting**: This state is entered when the waiting time of *Idle* state is elapsed and start command is sent to thrusters.
(3) **Moving**: The robot is moving, rotating, or station-keeping. This depends on the control strategy.
(4) **ChangeConfig**: This state is to be activated when the robot changes its configurations. In this state, the robot modifies the positions and orientations of thrusters, not doing anything else (i.e., another task).
(5) **Moving/ChangeConfig**: The robot enters this state when a command to change configuration is received. The robot is still performing current missions and changing its configurations.
(6) **Stopping**: The robot enters this state when the current mission is finished.

**Figure 6:**
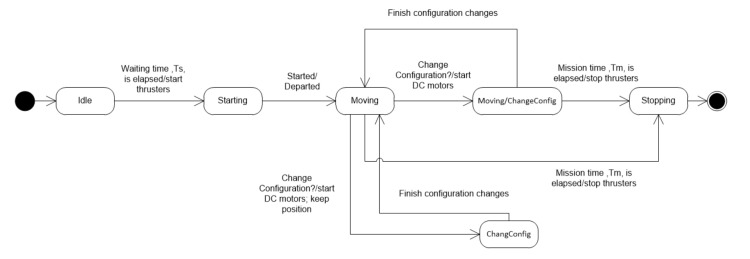
The dynamic state machine modeling of *Turn on power/automatic* use case.

Figure 6 shows the transitions between states. The notation *condition/action* is used to describe the transition arrows.

**Figure 7:**
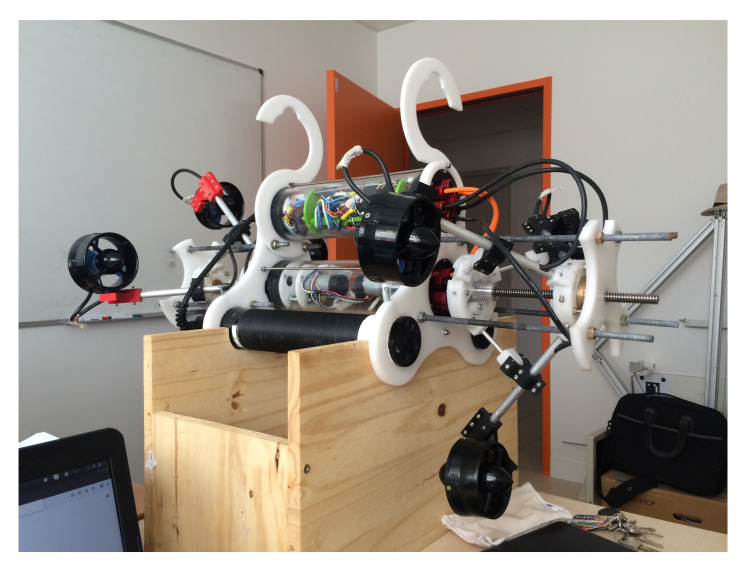
A prototype of Umbrella Robot https://www.youtube.com/watch?v=yBBCu1z3q-0&feature=youtu.be accessed on 8 February 2022.

The current version of Umbrella Robot at LIRMM, Montpellier University, is shown in Figure 7. For varying configuration, readers can follow a video link in Figure 7.

## 3. Reconfigurability

**Figure 8:**
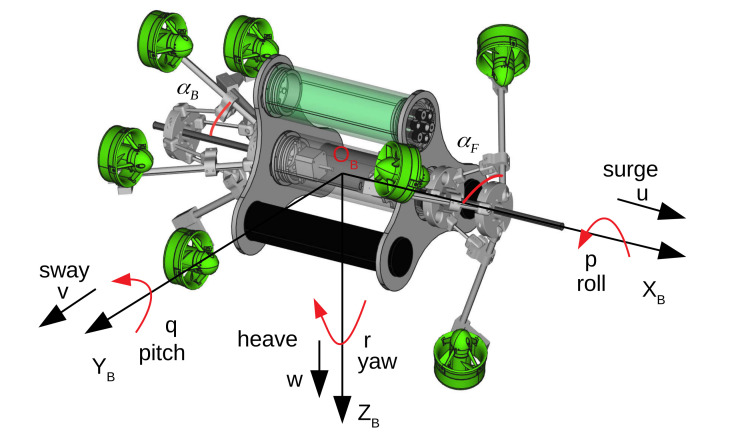
Definitions of two angles $\alpha_{F}$ and $\alpha_{B}$ and other notations.

The reconfigurability of the robot is expressed by modifying two angles $\alpha_{F}$ and $\alpha_{B}$ (see Figure 8); therefore, the configuration matrix and the A matrix will change accordingly. In fact, the relation between thrusters forces and resulting forces (forces/torques with respect to body frame) is usually computed as follows:

(1) FB=FΓ=A(αF,αB)Fm

where $F_{B}={\left[F_{u}\;F_{v}\;F_{w}\;\Gamma_{p}\;\Gamma_{q}\;\Gamma_{r}\right]}^{T}\in R^{6}$ is a vector of the resulting force in which $F={\left[F_{u}\;F_{v}\;F_{w}\right]}^{T}$ and $\Gamma ={\left[\Gamma_{p}\;\Gamma_{q}\;\Gamma_{r}\right]}^{T}$, $A\left(\alpha_{F},\alpha_{B}\right)\in R^{6\times m}$, the thruster force vector $F_{m}={\left[F_{m,1}\;F_{m,2}\;\ldots \;F_{m,m}\right]}^{T}\in R^{m}$ is a vector of the thruster’s forces, and *m* is the number of thrusters, m=7>6.

From the scheme of Umbrella Robot, the configuration matrix, A, has a form as:

(2) $$\begin{array}{c} \begin{array}{cc} A\; & =\left(\begin{array}{cccc} u_{1}^{B} & u_{2}^{B} & \ldots & u_{m}^{B}\\ r_{1}^{B}⊗u_{1}^{B} & r_{2}^{B}⊗u_{2}^{B} & \ldots & r_{m}^{B}⊗u_{m}^{B} \end{array}\right)\\ \; & =\left(\begin{array}{cccc} u_{1}^{B} & u_{2}^{B} & \ldots & u_{m}^{B}\\ \tau_{1}^{B} & \tau_{2}^{B} & \ldots & \tau_{m}^{B} \end{array}\right)=\left(\begin{array}{c} A_{1}\\ A_{2} \end{array}\right) \end{array} \end{array}$$

where m=7; $u_{i}^{B}$ and $r_{i}^{B}$(i∈{1,…,7}) are direction and position vectors of thrusters with respect to body frame. The operator ⊗ is a cross product, and $\tau_{i}^{B}=r_{i}^{B}⊗u_{i}^{B}$. The $u_{1}^{B},\ldots ,u_{7}^{B}$ and $r_{1}^{B},\ldots ,r_{7}^{B}$ are identified as in Appendix A.

From equations $u_{i}^{b}$ and $r_{i}^{b}$, it is obvious to see that the configuration matrix (A) depends on forward and backward angles, $\alpha_{F}$ and $\alpha_{B}$, respectively. By modifying these two angles, the configuration matrix will change. Table 1 shows the configuration matrix A corresponding to three cases. If $\alpha_{F}=\alpha_{B}=45^{\circ }$, the robot is propelled with all thrusters oriented in the same direction, called *torpedo configuration*, and it can act along surge, pitch, and yaw degrees of freedom. This configuration corresponds to an under-actuated situation, and the system can be controlled as a torpedo-like system. Otherwise, if $\alpha_{F}=\alpha_{B}=90^{\circ }$, the robot can act along the 6 DoFs, it is a fully actuated system (note that in the roll direction, the acting ability is quite small in our case); therefore, the acting capability of the robot is extended. In an arbitrary case, for instance $\alpha_{F}=50^{\circ },\alpha_{B}=60^{\circ }$, the configuration matrix also shows that the robot can operate along 6 DoFs; however, the priority is also along *u*-axis (surge direction), and then the robot can be considered as a fully actuated system, but with different capability along a specific DoF. If two angles $\alpha_{F}$ and $\alpha_{B}$ vary at each time step, the online adaption of the configuration matrix can be achieved.

### Acting Ability

From Equation (1), we can see that the acting abilities along each DoF depends on the elements of matrix $A\in R^{6\times m}$ (with elements $a_{ij}$); therefore, we define the *acting ability* criterion for each DoF as:

(3) $$\begin{array}{cc} K_{i} & =\sum_{j=1}^{m}a_{ij}^{2} \end{array}$$

where i∈{u,v,w,p,q,r}, specifically, *u*—surge linear velocity, *v*—sway linear velocity, *w*—heave linear velocity, *p*—roll angular rate, *q*—pitch angular rate, and *r*—yaw angular rate; $a_{ij}$ (row *i*, column *j*) is an element of matrix A. In particular, *acting ability* along *u*-axis is $K_{u}=\sum_{j=1}^{m}a_{uj}^{2}$, note that *u* corresponds to row-1, *v* corresponds to row-2, *w* corresponds to row-3, *p* corresponds to row-4, *q* corresponds to row-5, and *r* corresponds to row-6.

**Figure 9:**
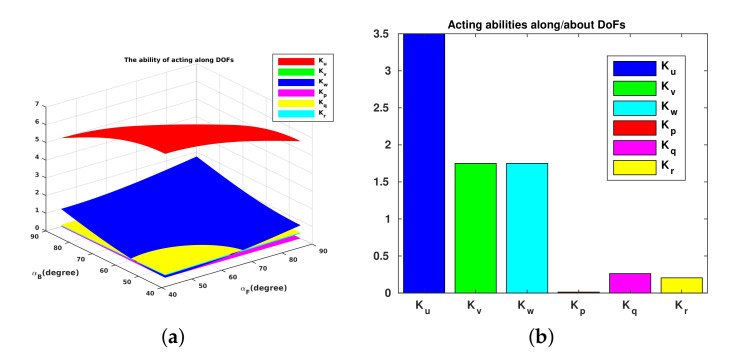
Acting abilities of UR. (**a**) Acting abilities along/about each DOF of UR with varying $\alpha_{F}$ and $\alpha_{B}$. (**b**) Acting abilities along/about each DOF of UR with $\alpha_{F}=\alpha_{B}=90^{\circ }$.

For Umbrella Robot, two angles $\alpha_{F},\alpha_{B}$ vary from $45^{\circ }$ to $90^{\circ }$. We can illustrate variations of these capabilities as shown in Figure 9a. We can obviously choose the maximum value of the acting ability of each DoF; however, there exists a large deviation of acting abilities between DoFs. In particular, the acting abilities of 6 DoFs with $\alpha_{F}=\alpha_{B}=90^{\circ }$ are shown in Figure 9b.

This Figure 9 shows the acting ability (which is defined in Equation (3)) of the robot for each Degree of Freedom (DoF) (surge, sway, heave, roll, pitch, yaw) when its configuration is changed. Specifically, Figure 9a presents the variety of acting ability when varying two angles ($\alpha_{F},\alpha_{B}$); Figure 9b presents the acting ability when these two angles equal $90^{\circ }$. One could note that the acting capability $K_{p}$ is very low. This is due to the fact that in the *open-open* configuration, all the thrusters are nearly crossing the *x*-axis of the system, thus inducing a very poor roll torque. Nevertheless, this degree of freedom can be statically compensated with a judicious placement of weighting parts (batteries) and buoyancy foam.

## 4. Experiments

**Figure 10:**
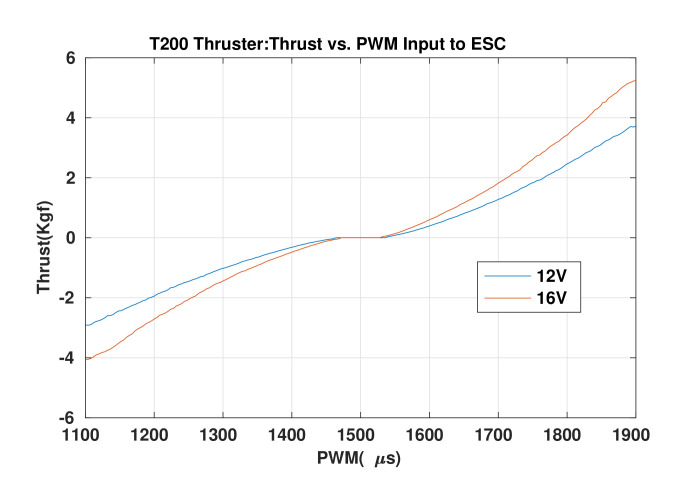
T200 thruster’s characteristics adapted from [32].

The objectives of the experiments were to validate the UR’s basic operations in each major DoF (i.e., surge, heave, yaw, pitch controls), and an integrated mission (a combination in different tasks) with its reconfigurability (changing its configuration during a mission). Note that the UR uses the T200 thruster, which has the characteristic shown in Figure 10, as the actuators. The input of the thruster is PWM (range from 1100 (μs) to 1900 (μs)) that is the input to the Electronic Speed Controller (ESC) inside; however, in our experiments, this was limited from 1200 (μs) to 1800 (μs) for safety. The output of the thruster is the force.

**Figure 11:**
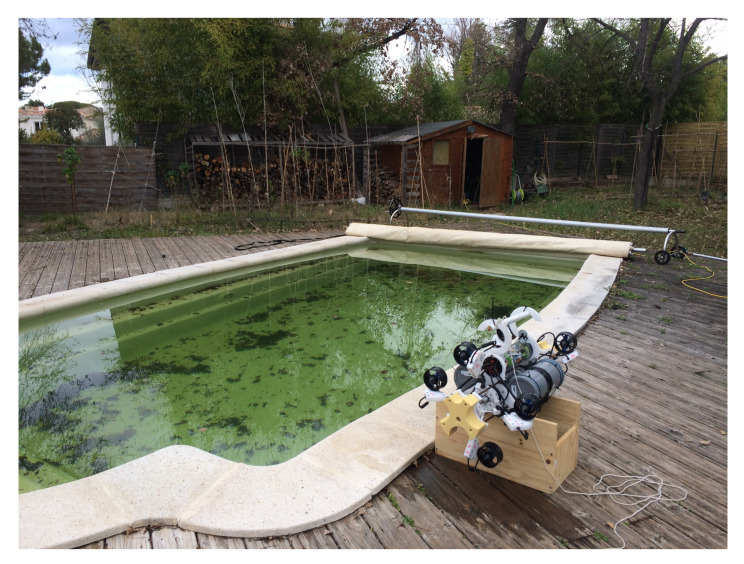
Umbrella Robot at the swimming pool.

The experiments were carried out in a swimming pool (see Figure 11) with specific missions, i.e., *yaw control, depth control, surge–pitch–yaw control, and finally an integrated mission*. The simple PD (Proportional Derivative) controllers were used in our experiments. The parameters of the controller are shown in Table 2.

### 4.1. Yaw Control

**Figure 12:**
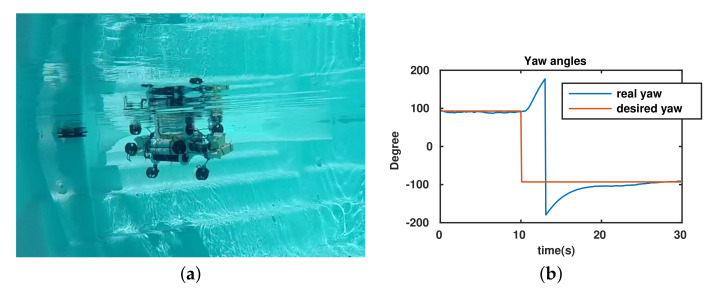

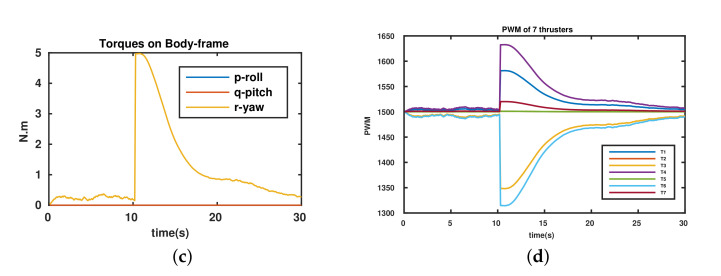
Yaw control of the UR. (**a**) Position and direction of thrusters of UR in the yaw control. (**b**) Yaw angles. (**c**) Applied torques. (**d**) PWM (μs) of thrusters.

In this experiment, the desired yaw angle is set to $93^{\circ }$ (initial yaw angle of the robot) and after to $-93^{\circ }$. The objective was that the UR maintains an initial yaw angle ($93^{\circ }$) and makes a turn to a second desired angle ($-93^{\circ }$). In this test, the robot’s configuration is chosen as $\alpha_{F}=\alpha_{B}=45^{\circ }$ (the torpedo configuration); therefore, the position and direction of the thrusters are as seen in Figure 12a. The experimental results are shown in Figure 12. In particular, the real yaw angle of robot follows the desired yaw angle as in Figure 12b. Moreover, applied torques, outputs from controller, and PWM (Pulse Width Modulation) inputs of the thrusters are presented in Figure 12c,d, respectively. When the UR keeps the initial yaw angle, from 0–10 s, the outputs of the controller are almost zero and all thrusters do not rotate. When the desired yaw angle is changed, the controller delivers yaw torques to converge to the desired value ($-93^{\circ }$), the time response of the system is around 15 s.

### 4.2. Depth Control

**Figure 13:**
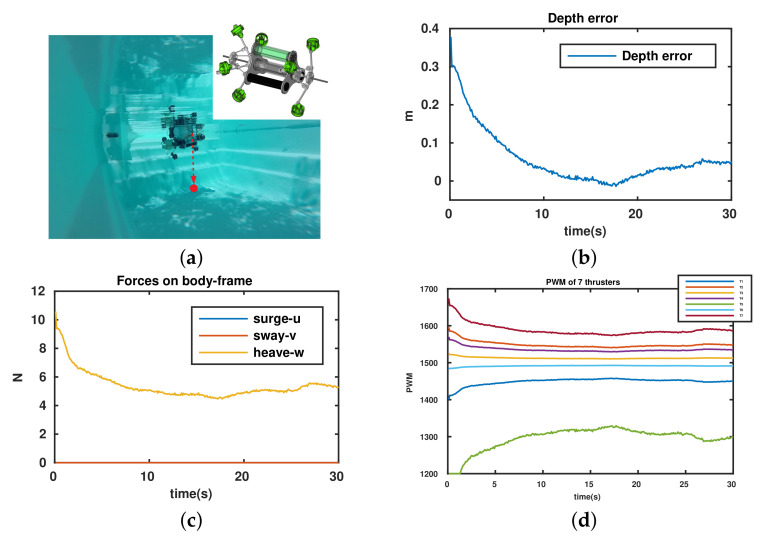
Depth control of the UR. (**a**) Mission description, position, and direction of thrusters in the depth control. (**b**) Depth error = desired depth–real depth. (**c**) Applied forces. (**d**) PWM (μs) of thrusters.

In this test, robot’s configuration is set as $\alpha_{F}=\alpha_{B}=70^{\circ }$ (the position and direction of all thrusters are shown in the right upper-corner of Figure 13a) and the desired depth is set as a constant. The experimental results are shown in Figure 13. The mission was to control the robot to a desired depth as in Figure 13a. The depth error, that is the deviation between desired depth and real depth, converges to zero and remains stable as shown in Figure 13b. Only the force applied along the heave direction (output from the controller) is delivered as in Figure 13c. The PWM inputs of thrusters remain in a feasible region, except for Thruster 5, which reaches saturation around 2 s (Figure 13d). We can see that a simple controller can be used to control the robot to a desired depth.

### 4.3. Surge–Pitch–Yaw Control

**Figure 14:**
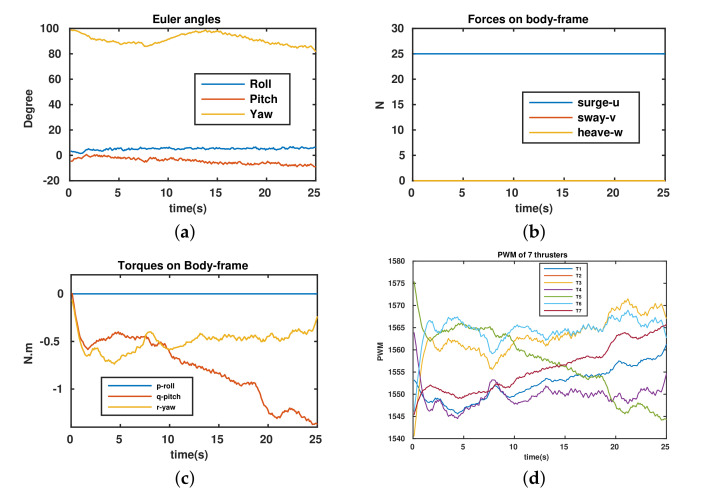
Surge, pitch, and yaw control https://youtu.be/1DzfYrsSaMM and https://youtu.be/9eFT7h-zX3s accessed on 8 February 2022. (**a**) Euler angles. (**b**) Applied forces. (**c**) Applied torques. (**d**) PWM (μs) of thrusters.

The objective of this experiment was to control the robot with different tasks, i.e., surge forward and maintain the pitch and yaw stability using the configuration as a torpedo-like shape. The robot’s configuration is set up as $\alpha_{F}=\alpha_{B}=45^{\circ }$. All thrusters have the same directions as in the experiment of yaw control; however, in this test, the robot has to maintain the desired pitch and yaw angles and to surge forward. Hence, the force $F_{u}=25$ N is applied along the surge direction (Figure 14b). The experimental results are shown in Figure 14. Readers can see more with attached video links. Figure 14a shows that the Euler angles of the robot (roll, pitch, and yaw) remain stable during the mission. Moreover, applied torques (around pitch and yaw) produced to keep the stability of these angles are shown in Figure 14c. The PWM inputs of the thrusters are kept inside the feasible region (Figure 14d).

### 4.4. An Integrated Mission

**Figure 15:**
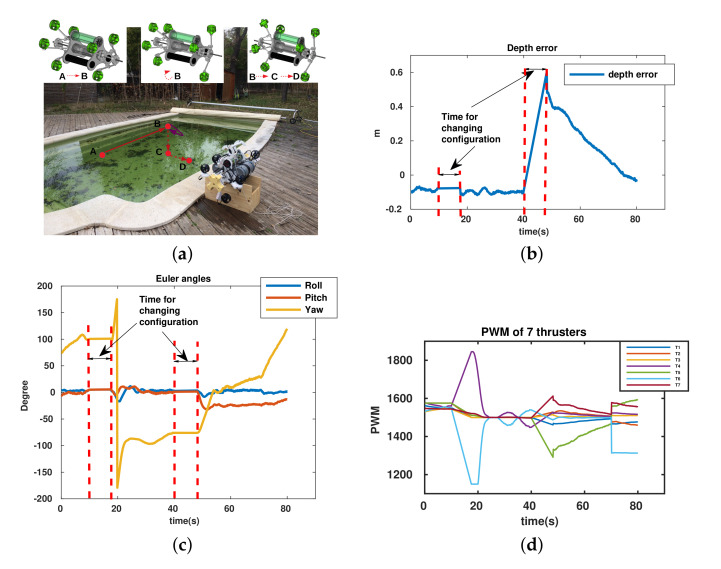
Integrated mission of Umbrella Robot. (**a**) Mission description, position, and direction of thrusters. (**b**) Depth error = desired depth–real depth. (**c**) Euler angles. (**d**) PWM (μs) of thrusters.

The final experiment proposes to validate the robot’s performance with different configurations during a mission. In fact, in this test, the robot carries out a complex task, which is illustrated in Figure 15a. Specifically, it goes straight from point **A** to point **B** (surge–pitch–yaw control) ($\alpha_{F}=\alpha_{B}=45^{\circ }$) (the position and direction of all thrusters are as seen in the left upper-corner of Figure 15a), and then it changes configuration with $\alpha_{F}=85^{\circ }$ and $\alpha_{B}=45^{\circ }$ (the position and direction of all thrusters are as seen in the upper center of Figure 15a) and makes a turn $180^{\circ }$ around point **B**. After that, it changes to a new configuration ($\alpha_{F}=85^{\circ }$ and $\alpha_{B}=85^{\circ }$) (the position and direction of all thrusters are as seen in the right upper-corner of Figure 15a) and performs a depth control in a fixed time from point **B** to point **C**. Finally, with the same configuration, the robot moves along the sway direction and maintains the depth control (see Figure 15a). The experimental results are shown in Figure 15. Note that the robot has to modify its configuration with respect to the task because of the directly uncontrollable command. For example, with $\alpha_{F}=\alpha_{B}=45^{\circ }$, the robot cannot be driven to a desired depth directly; it can be controlled indirectly by more complex strategy; however, it is not considered in the paper.

In this mission, the robot requires the time to change its configuration as in Figure 15b,c. It takes around 7 s and this time depends the power of the actuator being in charge of the change (DC motors). The control performance is achieved thanks to the convergence of depth error, the deviation between desired depth and real depth (depth control), and the change of yaw angle (making a turn) (see Figure 15b,c). Finally, it is easy to see that the PWM inputs of the thrusters are also in the feasible region. We can see that the UR can implement different tasks with different configurations.

Through all the above experiments, the UR shows the ability to control the 5 DoFs (i.e., surge, heave, sway, pitch, yaw) easily. For the roll-DoF, the acting ability of the robot is rather small and requires more effort to control the robot in this direction. Theoretically, for our robot, roll-DoF can be controllable (see Figure 9b); however, this was not implemented in the real test.

## 5. Discussion and Comparison

**Figure 16:**
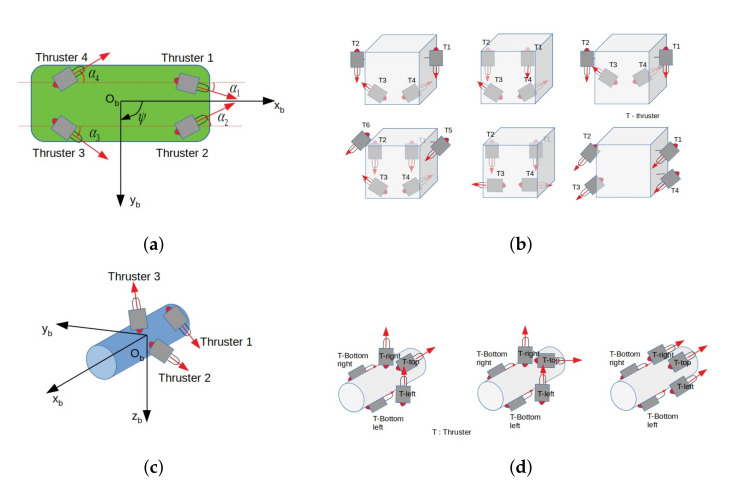
Different reconfigurable underwater robots. (**a**) Tortuga robot’s configuration. (**b**) e-URoPe robot ’s configuration. (**c**) RSM robot’s configuration. (**d**) Amour 6 robot’s configuration.

The Umbrella Robot (UR), a dynamically reconfigurable robot, has been proposed, designed, and tested. Generally, for a reconfigurable robot, its configuration can be changed statically or dynamically. It is obvious to see that a dynamic one is more versatile than a static one, especially for a challenging environment, such as a confined karst. In this section, we make a comparison between our robot and different reconfigurable underwater robots, i.e., *Tortuga* [33], *e-URoPe* [29], *RSM* [18], and *Amour 6* [34], which are shown in Figure 16. Note that, among them, only *Tortuga* and *RSM* are able to dynamically change configuration during a mission. For *e-URoPe* and *Amour 6*, several possible layouts can be adjusted statically depending on a specific mission. To this end, we assume that a DoF is called *controlled* if there exists at least one actuator that can deliver a force or torque directly along/about this DoF. Otherwise, it is called *uncontrolled*. A comparison between different kinds of robot on each DoF was carried out to see the versatility of each one. Thanks to our robot’s configuration and four others, the comparison is shown in Table 3. In particular, we can see that our robot, UR, is *controlled* in all DoFs. For the *Tortuga* robot, all DoFs are *controlled* except pitch DoF. For the *e-URoPe* robot, six possible layouts can be adjusted for the mission. Of course, for each layout, there exist several DoFs *uncontrolled* (i.e., roll and pitch). For *RSM* robot, theoretically, four thrusters can change orientation in all directions and all DoFs are *controlled*; however, there is no real test for this robot. Moreover, each thruster needs at least one actuator to drive, this raises the cost-effective issue and complexity in installation. The last robot, *Amour 6*, has three layouts in which pitch DoF cannot be *controlled*.

From a dynamic configuration perspective, we discuss our robot and *Tortuga*. Indeed, only our robot and *Tortuga* had real experiments in which the configuration had been changed during the mission. Following Figure 16a, the configuration matrix of the *Tortuga* robot depends on four angles ($\alpha_{1,2,3,4}$). For each thruster, one motor is needed to drive. The advantage of this mechanism is that each thruster can controlled separately; however, this raises the energy consumption, which is a problem for long-range missions and confined environments (e.g., karst). Otherwise, our robot only uses two added motors to change the direction and orientation of seven thrusters (see Table 4). This is a significant impact when we would like to design an over-actuated system that can be useful in the case of fault tolerance and endurance in long missions in confined environments. Furthermore, in order to evaluate a configuration design, we need performance criteria as in [31]. One of the important properties of the robot is its isotropic attribute, which means that the robot is able to act to the same degree in all DoFs. Based on the structure of the configuration matrices, our robot is more isotropic than the *Tortuga* robot. Of course, each robot’s design has been proposed for each priority.

## 6. An Application Case

The advantages of our robot’s flexibility can be clearly seen in the docking application. A docking system will enhance the endurance capability of AUVs for long-term missions while reducing operation costs and hazards. The functions of a docking station (DS) are normally for battery charging, and mission data download/upload without recovering the AUV back to a ship’s board. Various forms of docking stations have been developed; however, they can be classified into three popular implementations, either a *fixed* docking station, *free-floating* docking station, or *mobile* docking station. The first one is firmly fixed to the seabed or integrated into a subsea station; the second type is buoyant and moored to the seabed or connected to a larger underwater vessel. The last one is normally towed behind a ship [35].

**Figure 17:**
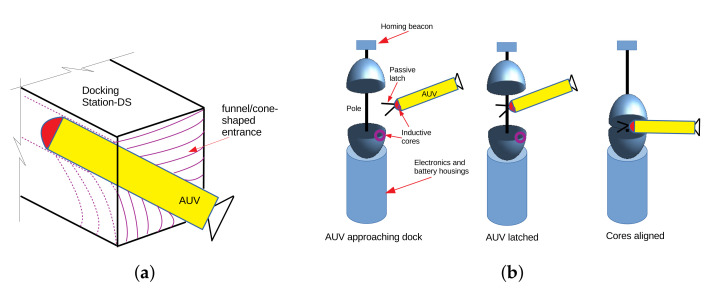
Mechanical configurations of DS types. (**a**) Unidirectional DS; (**b**) Omnidirectional DS.

Mechanical configurations of docking stations comprise the unidirectional DS or the omnidirectional DS (see Figure 17). The unidirectional DS is a capture mechanism that consists of a funnel/cone-shaped entrance for catching torpedo-shaped AUVs (Figure 17a) [36]. The omnidirectional DS is a vertical structure including rigid poles or cables under tension, to which a vehicle is able to attach using a latching device (Figure 17b) [37]. The key advantage of this DS is to allow an AUV to approach from any direction and a simple navigation system can be used during the docking task.

The docking procedure can be divided into two major stages, known as the *homing stage* and *docking stage*. In the *homing stage*, an AUV has a high-speed approach to arrive in the close vicinity of the DS. In the *docking stage*, the AUV has a low-speed final approach that requires the vehicle to be controllable at a low speed to drive it into the DS entrance and to latch it in the DS. Moreover, this will remove constraints on the nature of the docking structure, will achieve greater control of alignment of the vehicle and docking during capture, and minimize docking forces. This implies a vehicle with control over four or more degrees of freedom at low speed. Another simpler DS will be designed for this kind of vehicle. This idea is called *soft-docking* approaches [37]. In the literature, most of the docking stations have been designed for torpedo-shaped AUVs. This can be clearly seen in Table 5. In fact, all robots were designed as under-actuated systems with three controllable DoF. This requires more complexity in building navigation, guidance, and control system of robots as well as docking station. Moreover, the *soft-docking* approach cannot be applied for these robots.

**Figure 18:**
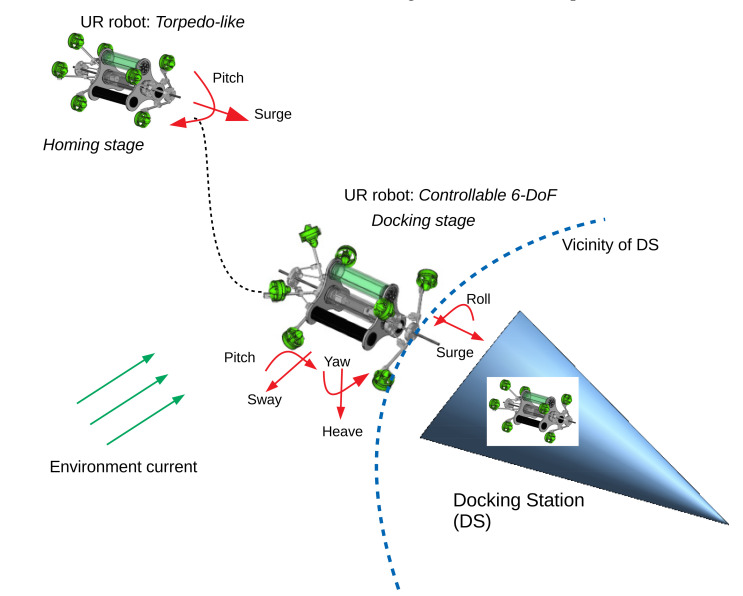
Concept of docking procedure of UR robot.

Motivated by the *soft-docking* approach, the UR’s application for the docking problem is proposed as in Figure 18, and can be summarized as follows:

(1) *Homing stage*: The UR uses a torpedo-like configuration to obtain a high speed to arrive close in the vicinity of the DS.
(2) *Docking stage*: The UR changes its configuration to become a 6-DoF controllable vehicle in order to approach and latch onto the DS easily and to deal with environment disturbances. It is clear that, with a 6-DoF controllable AUV, the navigation, guidance, and control for the AUV and the docking station will be simpler.

The advantages of the UR robot will enhance a docking solution thanks to its reconfigurability. It is similar to the application of the UR (Umbrella Robot) to karst exploration that was mentioned before. Indeed, the robot needs a high-speed approach with the torpedo-like configuration to overcome a narrow conduit and to go fast to the target and then requires a low-speed profile (6-DoF controllable) to make observations or to collect samples.

## 7. Conclusions and Future Works

The paper proposes a dynamically reconfigurable AUV that can modify its configuration with respect to different mission requirements. The hardware and software designs of the robot are presented, such as class modeling and dynamic state machine modeling. The reconfigurable capacity of the robot has been introduced and analyzed thanks to the variation of the configuration matrix. Experimental results are shown to demonstrate the versatility of the robot. The discussion and comparison between our robot and four other underwater robots are also presented, and an application case (docking problem) is suggested and discussed; however, the robot has a limitation in roll rotation. An optimal shape of the robot to improve this drawback will be an interesting work. Moreover, when investigating adaptation to the variation of the robot’s configuration, some challenging issues will be derived, such as an adaptive control strategy (i.e., switched controller between an under-actuated system and a fully/over-actuated system) and an adaptive control allocation. An energy-efficient path following, trajectory tracking, or docking implementation with the dynamically reconfigurable robot will be the subject of future research. Finally, we are looking forward to conduct experiments in a real karstic conduit.

## Figures and Tables

**Table:** 

	
(**a**)	(**b**)

(**c**)	

**Table:** 

	
(**a**)	(**b**)

**Table:** 

	
(**a**)	(**b**)

**Table:** 

	
(**a**)	(**b**)
	
(**c**)	(**d**)

**Table:** 

	
(**a**)	(**b**)
	
(**c**)	(**d**)

**Table:** 

	
(**a**)	(**b**)
	
(**c**)	(**d**)

**Table:** 

	
(**a**)	(**b**)
	
(**c**)	(**d**)

**Table:** 

	
(**a**)	(**b**)

**Table 1:** Configuration matrix with some cases of two angles $\alpha_{F}$ and $\alpha_{B}$.

Angles	*A* Matrix
$\alpha_{F}=45^{\circ }$ $\alpha_{B}=45^{\circ }$	$\left(\begin{array}{ccccccc} 1 & 1 & 1 & 1 & 1 & 1 & 1\\ 0 & 0 & 0 & 0 & 0 & 0 & 0\\ 0 & 0 & 0 & 0 & 0 & 0 & 0\\ 0 & 0 & 0 & 0 & 0 & 0 & 0\\ -0.0435 & 0.1909 & -0.0435 & -0.0600 & 0.1909 & -0.0600 & -0.1909\\ 0.1953 & -0.0600 & -0.1953 & 0.1909 & -0.0600 & -0.1909 & 0.0600 \end{array}\right)$
$\alpha_{F}=50^{\circ }$ $\alpha_{B}=60^{\circ }$	$\left(\begin{array}{ccccccc} 0.9962 & 0.9962 & 0.9962 & 0.9659 & 0.9659 & 0.9659 & 0.9659\\ 0.0755 & 0 & -0.0755 & 0.2588 & 0 & -0.2588 & 0\\ 0.0436 & -0.0872 & 0.0436 & 0 & -0.2588 & 0 & 0.2588\\ -0.0052 & -0.0052 & 0.0052 & 0.0155 & -0.0155 & -0.0155 & -0.0155\\ -0.0630 & 0.2296 & -0.0630 & -0.0580 & 0.1417 & -0.0580 & -0.1417\\ 0.2287 & -0.0598 & -0.2287 & 0.1417 & -0.0580 & -0.1417 & 0.0580 \end{array}\right)$
$\alpha_{F}=90^{\circ }$ $\alpha_{B}=90^{\circ }$	$\left(\begin{array}{ccccccc} 0.7071 & 0.7071 & 0.7071 & 0.7071 & 0.7071 & 0.7071 & 0.7071\\ 0.6124 & 0 & -0.6124 & 0.7071 & 0 & -0.7071 & 0\\ 0.3536 & -0.7071 & 0.3536 & 0 & -0.7071 & 0 & 0.7071\\ -0.0424 & -0.0424 & 0.0424 & 0.0424 & -0.0424 & -0.0424 & -0.0424\\ -0.1575 & 0.3885 & -0.1575 & -0.0424 & 0.0566 & -0.0424 & -0.0566\\ 0.3577 & -0.0424 & -0.3577 & 0.0566 & -0.0424 & -0.0566 & 0.0424 \end{array}\right)$

**Table 2:** Parameters of the PD controllers.

	$K_{p}$	$K_{D}$
Yaw control	10	2
Depth control	20	5
Surge–Pitch–Yaw control	10	3
Integrated Mission	15	4

**Table 3:** A comparison between different underwater robots.

	UR Robot	Tortuga	e-URoPe	RSM	AMOUR 6
Surge	Controlled	Controlled	Controlled	Controlled	Controlled
Sway	Controlled	Controlled	Controlled	Controlled	Controlled
Heave	Controlled	Controlled	Controlled	Controlled	Controlled
Roll	Badly Controlled	Controlled	UnControlled	Controlled	Controlled
Pitch	Controlled	UnControlled	UnControlled	Controlled	UnControlled
Yaw	Controlled	Controlled	Controlled	Controlled	Controlled
Reconfig	Dynamic	Dynamic	Static	Dynamic	Static

**Table 4:** Added actuators/changed thrusters comparison between UR and Tortuga.

	UR Robot	Tortuga
Added actuators	2	4
Changed thrusters	7	4

**Table 5:** Docking problem with different robots.

	Configuration	DS Type	Controllable DoF
REMUS [36] REMUS-100 [38] REMUS-600 [39]	Torpedo-shaped	Funnel-shaped DS	Surge, Pitch, Yaw
Odyssey IIB [40]	Torpedo-shaped	Fixed funnel-shaped DS	Surge, Heave, Yaw
Dorado [41]	Torpedo-shaped	Fixed funnel-shaped DS	Surge, Heave, Yaw
WL-3 AUV [42]	Torpedo-shaped	Fixed funnel-shaped DS	Surge, Pitch, Yaw
ISiMI [43]	Torpedo-shaped	Fixed funnel/cone-shaped DS	Surge, Pitch, Yaw
Ifremer Asterx [44]	Torpedo-shaped	Mobile funnel/cone-shaped DS	Surge, Heave, Yaw
Sparus II AUV [45]	Torpedo-shaped	Funnel/cone-shaped DS	Surge, Heave, Yaw

## Abbreviations

The following abbreviations are used in this manuscript:

AUV	Autonomous Underwater Robot
AS	Actuation System
S-NGC-A	Sensory Navigation Guidance Control Actuation
UR	Umbrella Robot
ROV	Remotely Operation Vehicle
IMU	Inertial Measurement Unit
PWM	Pulse Width Modulation
DoF	Degree of Freedom
ESC	Electronic Speed Controller
DS	Docking Station

## Appendix A

The elements of configuration matrix, A, are presented in Table A1. Note that *d* and $d_{i},i\in \left\{F,e,t\right\}$ are geometrical distances corresponding with the shape of the robot. $\beta_{i},i\in \left\{1,2,\ldots ,7\right\}$ is an angle.

**Table A1:** Elements of **A** matrix.

$u_{1}^{B}=\left(\begin{array}{c} \frac{1}{\sqrt{2}}\left(sin\alpha_{F}+cos\alpha_{F}\right)\\ \frac{\sqrt{3}}{2\sqrt{2}}\left(-cos\alpha_{F}+sin\alpha_{F}\right)\\ \frac{1}{2\sqrt{2}}\left(-cos\alpha_{F}+sin\alpha_{F}\right) \end{array}\right)$	$r_{1}^{B}=\left(\begin{array}{c} \left(d_{F}+L_{F}\right)-\sqrt{d_{e}^{2}+d^{2}}.cos\left(\alpha_{F}+\beta_{1}\right)\\ -\frac{d_{t}}{2}-\frac{\sqrt{3(d_{e}^{2}+d^{2})}sin\left(\alpha_{F}+\beta_{1}\right)}{2}\\ \frac{\sqrt{3}}{2}d_{t}-\frac{\sqrt{d_{e}^{2}+d^{2}}sin\left(\alpha_{F}+\beta_{1}\right)}{2} \end{array}\right)$
$u_{2}^{B}=\left(\begin{array}{c} \frac{cos\alpha_{F}+sin\alpha_{F}}{\sqrt{2}}\\ 0\\ \frac{cos\alpha_{F}-sin\alpha_{F}}{\sqrt{2}} \end{array}\right)$	$r_{2}^{B}=\left(\begin{array}{c} \left(d_{F}+L_{F}\right)-\sqrt{d^{2}+d_{e}^{2}}cos\left(\alpha_{F}+\beta_{2}\right))\\ d_{t}\\ \sqrt{d^{2}+d_{e}^{2}}sin\left(\alpha_{F}+\beta_{2}\right) \end{array}\right)$
$u_{3}^{B}=\left(\begin{array}{c} \frac{1}{\sqrt{2}}\left(sin\alpha_{F}+cos\alpha_{F}\right)\\ \frac{\sqrt{3}}{2\sqrt{2}}\left(cos\alpha_{F}-sin\alpha_{F}\right)\\ \frac{1}{2\sqrt{2}}\left(sin\alpha_{F}-cos\alpha_{F}\right) \end{array}\right)$	$r_{3}^{B}=\left(\begin{array}{c} \left(d_{F}+L_{F}\right)-\sqrt{d_{e}^{2}+d^{2}}.cos\left(\alpha_{F}+\beta_{3}\right)\\ \frac{d_{t}}{2}+\frac{\sqrt{3(d_{e}^{2}+d^{2})}}{2}.sin\left(\alpha_{F}+\beta_{3}\right)\\ \frac{\sqrt{3}}{2}d_{t}-\frac{\sqrt{d_{e}^{2}+d^{2}}}{2}sin\left(\alpha_{F}+\beta_{3}\right) \end{array}\right)$
$u_{4}^{B}=\left(\begin{array}{c} \frac{cos\alpha_{B}+sin\alpha_{B}}{\sqrt{2}}\\ \frac{-cos\alpha_{B}+sin\alpha_{B}}{\sqrt{2}}\\ 0 \end{array}\right)$	$r_{4}^{B}=\left(\begin{array}{c} -(d_{F}+\sqrt{d^{2}+d_{e}^{2}}cos\left(\alpha_{B}+\beta_{4}\right))\\ -(\sqrt{d^{2}+d_{e}^{2}}sin\left(\alpha_{B}+\beta_{4}\right))\\ -dt \end{array}\right)$
$u_{5}^{B}=\left(\begin{array}{c} \frac{cos\alpha_{B}+sin\alpha_{B}}{\sqrt{2}}\\ 0\\ \frac{cos\alpha_{B}-sin\alpha_{B}}{\sqrt{2}} \end{array}\right)$	$r_{5}^{B}=\left(\begin{array}{c} -(d_{F}+\sqrt{d^{2}+d_{e}^{2}}cos\left(\alpha_{B}+\beta_{5}\right))\\ d_{t}\\ \sqrt{d^{2}+d_{e}^{2}}sin\left(\alpha_{B}+\beta_{5}\right) \end{array}\right)$
$u_{6}^{B}=\left(\begin{array}{c} \frac{cos\alpha_{B}+sin\alpha_{B}}{\sqrt{2}}\\ \frac{cos\alpha_{B}-sin\alpha_{B}}{\sqrt{2}}\\ 0 \end{array}\right)$	$r_{6}^{B}=\left(\begin{array}{c} -(d_{F}+\sqrt{d^{2}+d_{e}^{2}}cos\left(\alpha_{B}+\beta_{6}\right))\\ \sqrt{d^{2}+d_{e}^{2}}sin\left(\alpha_{B}+\beta_{6}\right)\\ -d_{t} \end{array}\right)$
$u_{7}^{B}=\left(\begin{array}{c} \frac{cos\alpha_{B}+sin\alpha_{B}}{\sqrt{2}}\\ 0\\ \frac{-cos\alpha_{B}+sin\alpha_{B}}{\sqrt{2}} \end{array}\right)$	$r_{7}^{B}=\left(\begin{array}{c} -(d_{F}+\sqrt{d^{2}+d_{e}^{2}}cos\left(\alpha_{B}+\beta_{7}\right))\\ -d_{t}\\ -(\sqrt{d^{2}+d_{e}^{2}}sin\left(\alpha_{B}+\beta_{7}\right)) \end{array}\right)$
